# Nurses striving to provide caregiver with excellent support and care at a distance: a qualitative study

**DOI:** 10.1186/s12913-019-4740-7

**Published:** 2019-11-27

**Authors:** Hilde Solli, Sigrun Hvalvik

**Affiliations:** 1 Department of Nursing and Health Sciences, Faculty of Health and Social Sciences, University of South-Eastern Norway, Porsgrunn, Norway; 2 Kongsberg, Norway

**Keywords:** Caregiver, CMC, Home care services, Nurse, Telecare, Web camera, Web forum

## Abstract

**Background:**

In Norway, changes in life expectancy have led to increased attention to older people who are ageing at home, by means of home care services, adapted technology and informal caregivers. The caring situation has become difficult for many caregivers. The use of telecare has now offered them the possibility to receive support at home. The purpose of this study was to explore how nurses provide support and care at a distance, using a web camera and a web forum in a closed telecare network for caregivers to persons suffering from stroke and dementia.

**Methods:**

The study had an explorative design with a qualitative approach. The data sources consisted of interviews with nurses and excerpts from posts in a closed telecare network. Content analysis was used to analyse the text from the interviews and the text from the web forum.

**Results:**

The main theme, “Balancing asymmetric and symmetric relationships” described nurses’ relationship with caregiver. Two categories, “Balancing personal and professional qualities” and “Balancing caregivers’ dependence versus independence” were identified. The first describing the tension in their dialogue, the second describing how nurses provided the caregivers with a sense of security as well as strengthening them to master their daily lives.

**Conclusions:**

The nurses provided long distance support and care for the caregivers, by using computer-meditated communication. This communication was characterized by closeness as well as empathy. To strengthen the caregivers’ competence and independence, the nurses were easy accessible and provided virtual supervision and support. This study increases the knowledge about online dialogues and relationship between nurses and caregivers. It contributes to knowledge about balancing in the relationship, as well as knowledge about bridging the gap between technologies and nursing care as potential conflicting dimensions. Maintenance of ethical principles are therefore critical to be aware of.

## Background

In Norway, changes in the average life expectancy have led to increased attention paid to older people who are ageing at home, by means of home care services, adapted technology and informal caregivers [1–3]. Using information and communication technology (ICT) in healthcare services has made it possible to transmit voices, text and pictures in real and delayed time, hence providing computer-mediated communication (CMC) between persons. One way to practise this communication between nurses and patients or caregivers is by using telecare, defined as “[...] the use of information, communication, and monitoring technologies which allow healthcare providers to remotely evaluate health status, give educational intervention, or to deliver health and social care to patients in their homes” [4].

Informal caregivers perform an important function in home care service; therefore, it is essential to focus on their situation [5–7]. The literature reveals that the role of the caregiver may be difficult and impose physical and mental burdens [8]. Mental burdens are described as feelings of guilt, inadequacy, anger, sorrow, pain, emptiness, powerlessness and worry [9, 10]. The physical burden of always having to be the organiser of practical work is described as burnout, exhaustion and social dysfunction [6, 8, 10, 11]. Older caregivers often do not have anybody to talk to at home. Their need for somebody to talk too, might therefore be crucial. Some people may be homebound and socially isolated due to their caring roles [10, 12]. The use of telecare has now offered these persons the possibility of receiving support at home through communication with health professionals [4, 13, 14].

One of the core competencies in nursing practice is the capacity to establish good relationships with patients and relatives [15]. Orem, Taylor, and Renpenning [16] state that the nurse–patient relationship is complementary. This emphasises the significance of continuity in the collaboration between nurses and patients. The nurse–patient relationship is also essential for how to fulfil the patients’ needs. Travelbee [17] stresses the importance of person-centred care by focusing on the importance of perceiving the other person as a human and establishing a human-to-human relationship. The relationship is a reciprocal process in which relational development changes through the phases of the nurse–patient connection. However, the nurse is responsible for establishing and maintaining the relationship [17]. In addition, empathy is a core value in nursing. This means having the ability to enter into a process wherein one is able to precisely sense another’s inner experience at a given point of time [17].

To establish a relationship, the main interaction is through face-to-face communication [15, 17–19]. The nurse and the other person communicate via their appearances, behaviour, postures, facial expressions, mannerisms and gestures, whether or not they are aware of it [17]. In telecare, interpersonal communication and interaction are still present. However, the communication has changed from face-to-face in the same room to virtually face-to-face or by means of written messages. This new way of communicating might be to the benefit of patients as it facilitates access to healthcare providers. However, this type of communication also poses some challenges compared with traditional face-to-face communication. It is vital to consider these challenges.

Exploring the literature shows that video technology, text messages and health monitoring are the most commonly used forms of ICT in homecare services, although they are generally used on a small scale [20]. Only to a limited extent, have nurses used video consultation over a longer period to communicate with individual caregivers. Two studies describe this type of health care service [14, 21]. These studies maintain that web camera communication between health care personnel and caregivers is very important to caregivers in terms of technical, mental, and social support. In both studies, the caregivers experienced personal or close relationships with the health care personnel [14, 21]. However, one of the studies reports that some caregivers perceived virtual communication as strange or unnatural [14]. There is also a paucity of studies exploring the use of text messages between nurses and caregivers in home care services. Examination of the literature reveal three studies that dealt with the established ComputerLink health care service in the USA [22–24]. These studies describe how the nurse, as a moderator, gave textual support to the caregiver group, such as psychosocial support [24]. In addition, Brennan et al. [22] describe nurses’ ability to support caregivers in technical ways and explored how the service benefitted the caregivers [23].

More studies focus on nurses’ limited use of this communication tool due to their need for skill development with these technologies [25–27]. When new technology is adapted, nurses need to acquire knowledge of how this technology might expand or limit their nursing practice and the lives of their patients and the patients’ families. Nurses need to gain experience and knowledge of how a new practice influences their own lives and those of the persons for whom they care so that they can choose the best method of interaction.

## Methods

### Aim

This study aimed to explore how nurses in a nurse–caregiver relationship provide long-distance support and care using a web camera and a web forum in a closed CMC network.

### Design

We used an exploratory design with a qualitative approach. Explorative research is open and has little structure, offering the interviewer the possibility to follow-up with the interviewee to seek new information on the subject’s manifest and underlying processes [28, 29]. A qualitative approach is also suitable for revealing people’s own experiences, attitudes, thoughts or expectations about a phenomenon through interviews and texts. Moreover, this approach has the ability to shed light on interactions that have previously been only rarely described [30], such as the use of a web camera and web forum communication between nurses and caregivers.

### Setting

This project was linked to a public health care service for caregivers of people with dementia or who are stroke survivors. The supportive network service was implemented in three municipalities in a county in southern Norway and had operated for 5 years prior to the start of the study. The service package included four elements, with the intention that every participant should use the whole package when applying for the service. The service package comprised the following services:

1) A call centre was the headquarters for the three municipalities included in the network service. Several hours 2 days a week a part-time call centre nurse administered the call centre. The call centre nurse had the ability to log into the site with a web camera and have face-to-face communication with caregivers. She was also responsible for follow-up the caregivers to provide technical, mental and practical support. Likewise, the caregivers had the ability to contact the call centre nurse.

2) A digital information platform about diagnoses, symptoms, civil rights and advice constituted a repository of supportive knowledge. The call centre nurse provided updates and quality assurance regarding the knowledge.

3) Social meetings were held in the caregivers’ municipality, organised by a part-time municipality nurse. The main duty of the municipality nurse was to invite and organize social meetings, recruit new caregivers, give computer lessons to new participants, read and write in the web forum and follow-up with caregivers in their own municipality, either by ordinary phone or by web camera.

4) The web forum was a shared virtual room for all the caregivers and nurses included in the network service. In this web forum, they contributed written information, discussions, support, experiences or whatever the caregivers and nurses wanted to share.

This study was limited to the use of the web camera (1) and the web forum (4) between nurses and caregivers.

### Data sources

The participants were purposively selected. Four nurses and two auxiliary nurses (hereafter called nurses) comprised the entire staff in the network service. The nurse in charge asked the staff members to participate in the study. The participants were thoroughly informed about the aim of the study and about the researcher verbally as well as in written form prior to the study. They all returned a signed document of informed consent to the researcher [31].

Of the six nurses who were included in this study, three female nurses were the call centre nurses. One male and two female nurses were the municipality nurses. They all worked part-time and were aged 44 to 70 years old. The nurses’ duration of permanent employment in the network varied from 1 month to two and a half years at the time when they were included in this project. None refused to participate. However, two of the nurses finished their jobs after the first interview was held, and they therefore dropped out of the study.

The web forum was an electronic room in which all the participants in the network (approximately 44 caregivers and six nurses) could communicate via text. Every contribution was linked to a picture of the contributor. Initially, all participants who were connected to the network had agreed to make themselves available for research and that the content of the web forum could be used for research purposes.

### Data collection

The first author, HS, is a female registered nurse who is well versed in nurse and caregiver roles but had no prior experience using CMC as a means of communication in such a setting. Her occupation at the time of the study was academic research. HS conducted two individual interviews with four of the participants at their workplace. The two interviews were conducted to ensure that the participants had time to become familiar with the technology and thus could provide us with rich data. Two nurses were interviewed only once as they left the network. The first interview took place approximately one to 2 months after their inclusion in the study, and the second occurred approximately 6 months after inclusion.

We prepared an interview guide with semi-structured questions, which was pilot tested with one person before the study started. The questions about the use of the web camera were the following: “How would you describe your contact with the caregiver when you are using the web camera?” and “Can you tell me what you are talking about when you are using the web camera?” In the second interview, another question was asked: “Could you please tell me about the contacts you have had with caregivers over the last six months; have these changed in any way?” Likewise, the questions about the use of the forum were as follows: “Will you please tell me how you communicate with caregivers on the forum?” and “Could you tell me what discussions you join on the forum?” In the second interview, the following question was asked: “How would you describe your contacts with caregivers throughout the last six months; have these changed in any way?” These questions offered the possibility for follow-up to each interviewee’s answers and the opportunity to ask for elaboration when needed [32]. The interviews lasted for approximately 30–90 min each and were recorded and transcribed verbatim. HS made field notes just after the interviews for background information. These notes were not analysed but might have influenced the interpretation of the data.

The nurse in charge provided monthly online forum transcripts. In the beginning of the data collection period, all types of posts, regardless of their subjects, were extracted to ensure that we gained a broad understanding of the web forum content. During the process, we obtained many posts where caregivers wrote about non-care related activity such as comments about the weather. We then focused our approach on dialogue between caregivers and nurses to examine more closely how the nurses asked questions and answered the caregivers.

Over 21 months, we obtained 476 posts. Based on the collected posts and the individual interviews, further data was considered unnecessary as no new information was added. Thus, we considered that saturation was obtained [33].

### Analysis

We used a qualitative content analysis, inspired by Graneheim and Lundman [34]. The data were systematically broken down to small units and analysed and grouped into categories or themes based on their content [35].

The first author read and reread the texts from the interviews several times to be familiar with the content before it was organised in the proper meaning-based units. The meaning-based units were condensed and labelled with codes. All codes were grouped and sorted into two preliminary categories and five sub-categories. The same process was conducted with the text from the web forum. NVIVO 10 software [36] was used to facilitate the organisation of codes extracted from the web forum text into preliminary categories. Then, we merged the analysis of the interview text and the analysis of the web forum text into one analysis with the same categories and sub-categories. Finally, we abstracted and renamed the preliminary categories and sub-categories. The level of the categories and the sub-categories is regarded as the manifest content. During the analytical process a theme emerged from the data as the highest interpretive level. The theme shows a thread of latent meaning through condensed meaning units, codes and categories [34, 37]. However, it is not always possible to create mutually exclusive categories. A theme might therefore consist of multiple meanings developed within the categories and cutting across the categories to make both obvious and underlying meanings visible.

### Rigour

We considered the trustworthiness of the study in light of four criteria: credibility, confirmability, transferability, and auditability [34, 35, 38, 39].

To enhance credibility, we described in detail the study design, setting, data sources, data collection and analysis to show how well the data and process addressed the intended aim of the study. We formulated categories and structured the results to reflect the participants’ experiences, feelings and descriptions of the nurse-caregiver relationship. To establish confirmability, the co-author also examined the documentation of the analysis. The authors discussed the names of the terms. This process went back and forth until an agreement was reached about categories, sub-categories and the theme. In this way, we emphasized objectivity and ensured that the data represented the information from the informants. To promote transferability, we described the setting and the available data sources and gave a rich description of the findings. To ensure auditability, we explained the rationale for the logical decisions and choices we made throughout the process and explained the methodological decisions and choices we made during the research process.

## Results

Table 1 depicts the theme and subthemes. One main theme, Balancing asymmetric and symmetric relationships, emerged from the analysis. This theme depicted the nurses’ experiences of how they provided support and care in their nurse–caregiver relationships.

**Table 1:** Organisation of the results

Theme	Balancing asymmetric and symmetric relationships	
Category	Balancing personal and professional qualities	Balancing caregivers’ dependence versus independence
Sub-category	-Closeness -Empathy	-Accessibility -Strengthening the caregivers’ competence -Supervising

### Balancing asymmetric and symmetric relationships

The way the nurses described how they provided telecare revealed that the relationship between nurse and caregiver was asymmetric, as the nurse possessed professional competence that the caregivers needed. The nurses were employed in this healthcare service, with the responsibility to support caregivers in caring for their spouses. By virtue of their knowledge, they were the professionals with the aim of strengthening the caregivers’ self-care. As one of the nurses expressed, “As a call centre nurse, you need to have a special focus on preventing and relieving the caregivers’ sense of loneliness” (Call centre nurse). Therefore, they organised education and training for new caregivers so they could master the use of the new technology. Likewise, they supported and supervised the caregivers in handling their situations, both psychologically and practically. The nurses also served as the moderators of the web forum. They also provided information and had an overview of the total activity on the forum. On the web forum, the nurses motivated the participants to share their experiences and engage in discussions with other caregivers. The intention was that the group should develop a sense of being supported as well as to support and thereby strengthening their identity.

The symmetric relationship was revealed when the nurses co-operated with some of the experienced caregivers. These caregivers acted as peer supporters in teaching the new caregivers how to use the technology and be part of the network. Thus, they acted as the nurses’ equals. A nurse explained their co-operation, “In cases in which new caregivers have special challenges, the peer supporter and the call centre nurse discuss a strategy for the best way of supporting the person” (Call centre nurse). The nurses also described the way in which they shared expressions, personal experiences and humour, which indicated a more symmetric relationship built on confidence and trust.

These asymmetric and symmetric relationships appeared in a dynamic process, adapting to the content of the written or verbal dialogue with the caregivers.

#### Balancing personal and professional qualities

This category revealed how the nurses balanced their personal characteristics and professional knowledge in their attempt to establish a positive, long-distance relationship with caregivers. Their descriptions demonstrated how they used their qualities in interpersonal dialogues with caregivers. This meant presence and closeness in dialogues with an empathic and supportive approach. At the same time, they described how they used a professional approach in balancing their emotions and nursing knowledge, which implied an attitude of empathy towards caregivers in the dialogues.

### Closeness

The nurses expressed an experience of closeness and that they became influenced by this atmosphere when they met the caregiver on the web camera. Such meetings strengthened the feeling of closeness, and they had a sense of being present in the other person’s home. All the call centre nurses stated that the camera provided a more intimate relationship than could ever be delivered by phone. One of the nurses expressed it as follows:

When I saw this man sitting there with these headphones because of impaired hearing, wow, I thought, “It’s brilliant to be in this network when you have that kind of handicap”. He had not told me about it. In addition, there he was, sitting and talking to me when I was in my office, watching his context, while his spouse was resting in the other room. Hence, I got another impression of him (Call centre nurse).

The content in web-camera communication addressed many subjects, such as diseases, different kinds of frustration and anger, everyday life, memories from childhood or excursions. However, the nurse and the caregiver could also just be silent together. One nurse said, “One of the caregivers has a beautiful view, and sometimes, we just sit and look at the view together” (Call centre nurse).

Some of the nurses mentioned that when they looked into the other person’s eyes, they often observed signs of sadness. By listening to the caregiver and giving the person time to tell his or her story, they confirmed that they cared for the person and provided personal and professional closeness through the on-camera dialogue. One nurse noted, “It isn’t something I believe; I know. To be present as a listening nurse when the other person has an ill spouse with dementia – it’s caring” (Municipality nurse). In the nurses’ experiences, the caregivers opened up and placed their confidence in the nurses soon after first meeting, and the nurse–caregiver relationship rapidly developed. Through their virtual presence, they simulated closeness by giving each other an electronic hug by rubbing themselves on the cheek and sending it like a blown kiss.

The forum was the place for written dialogue. Reading contributions from the caregivers offered the nurses the possibility to get to know them well. Many of the caregivers were attentive to one another, exchanged thoughts, asked important questions about one another’s lives and shared humorous anecdotes. A call centre nurse could answer their questions but also ask for their points of view, experiences of the healthcare service, vacations or knowledge of issues to activate a discussion. Humour was an important ingredient in dialogues, for example, “Many thanks that you managed to fix it for me! I did promise to gild you if you managed. Now I have [a] problem with how to do it, so it has to be in [a] praise report, I’m afraid. Hugs from NN” (A caregiver). The call centre nurse answered, “Forget the gilding ; it’s too much stress and too much gilding stuff is needed! Talk to you later” (Call centre nurse).

### Empathy

The interviews provided insight into how the call centre nurses provided empathy to the caregivers in a professional way. They described the significance of offering the caregivers the opportunity to vent their frustrations in particular situations. As an example, they told that some spouses suffering from dementia became jealous when the caregiver was talking with somebody on the web camera. Such circumstances became a problem for some of the caregivers and resulted in some being unsure if they could continue to be a part of the network. The call centre nurses described how they tried to handle such complex situations, striving to support the caregivers in an empathic way. As one call centre nurse expressed:

I can just support her; it was what I tried to do when I said, “Maybe you should move your computer away from your dining room to have a private place for yourself and not be sitting close to your husband all the time. You have the right to do so, and he is able to sit alone in front of the TV or fireplace for a while” (Call centre nurse).

In this way, the nurse tried to support the caregiver by suggesting that she could make a private space for herself, gain more control over her life, interests and time and take care of her own needs. Supporting caregivers in their daily life and in crisis was important to reduce their feeling of loneliness and give them the strength to keep on going:

I believe it is support along the way [that] might reduce the feeling of loneliness. The caregivers are doing fabulous work with their spouses, but when they don’t have anyone there with the same kind of experience, then no one really understands what they are going through and what their despair comprises. Therefore, the most important [help] is to give them the feeling of being understood (Municipality nurse).

The nurses revealed their empathy for the caregivers in their attitudes towards the work. Many of the nurses stated that it was important to show empathy, to listen carefully and to be keenly aware of their duty of confidentiality when they worked with people experiencing a difficult life. Perceiving each person as unique and individualising care required the nurses to reflect on their own personal and professional experience. They underscored the importance of having personal life experiences, as one of the nurses expressed:

He had a chronically ill grandchild; he was devastated. If I didn’t have an experience of having chronically ill children or hadn’t experienced something sad in my life, then I [would have] done him no good. In this job, you need personal experience as well (Call centre nurse).

Over time, they came to know the caregivers well. It was important to maintain a close relationship; however, it was important to carefully follow the ethical guidelines, show empathy and not become too personal in their relationship. All the nurses admired the caregivers for keeping their spouses at home. They were sure that a significant reason was the caregivers’ ability to be participants in the network. They were also certain that an electronic communication network was an important nursing tool now and in the future.

One of the nurses described the CMC network in this way:

It is a supplement, and it is a service. But if a caregiver is able to care for his spouse at home, and if it can prevent a bad situation and can have the function of being a security alarm for caregivers, then I am positive – but we have to think ethically all the way (Municipality nurse).

Through this statement, she also stressed that an ethical evaluation should be an underlying principle concerning all the activities in the network.

#### Balancing caregivers’ dependence versus independence

The nurses tried to improve the caregivers’ knowledge, skills and self-care to strengthen their competence and independence. The nurses provided practical training in ICT, easily accessible information and supervision. Additionally, they challenged the caregivers to be more conscious of their own needs in different situations. Another important nursing dimension was to be virtually visible and easily accessible to provide a sense of security for the caregivers.

### Accessibility

Two days a week, the call centre nurse was accessible for web-camera dialogue with the caregivers. Her main duty was to get in touch regularly with each participant, either on the web camera or by phone. One municipality nurse was also sometimes accessible on the web camera, although it was not her primary task. She appreciated the dialogue and wanted to challenge the caregivers in her group to use the web camera more often.

In the second interview, one municipality nurse had been more accessible and active on camera with her group of caregivers and even with the caregivers from other municipalities. The new call centre nurse had started more frequent follow-ups with new caregivers, either on camera or by phone. As soon as the new caregivers logged on the website with the camera, she got in touch with them, encouraging them to access it frequently in the beginning until they became familiar with using the camera. Thereafter, she got in touch with them more regularly.

Though the municipality nurses had part-time jobs in the network, they more or less logged onto the forum every day. The new call centre nurse logged onto the forum daily, regardless of whether she was at the call centre. If somebody seemed to have trouble, she took care of it at once. All nurses considered the forum to be their main means of communication with the caregivers. If one of the caregivers wanted to ask for something or to get in touch with one of the nurses, they wrote it on the forum. When the nurses wanted to ask for the caregivers’ views about something or the municipality nurses invited their groups to social meetings, they wrote these messages on the forum, for example, “I’m back again, and I’m inviting you to come together Wednesday the 8^th^ of October at 12:00 pm. We don’t have anything special on the agenda; anyway, it’s nice to meet you all again. Who is coming?”(Municipality nurse).

### Strengthening the caregivers’ competence

The primary task of the municipality nurses was to strengthen the caregivers’ competence in adapting to the technology. They provided new caregivers with computer training and taught them how to use the forum and the web camera. They often co-operated with an experienced caregiver to demonstrate the web-camera dialogue or asked a caregiver to be a supporting peer for a new caregiver. The nurses experienced computer training as a valuable starting point for relational development. They were flexible in helping the caregivers on the phone, via the web camera, by posting on the web forum or even in their homes when technical problems occurred.

Posting messages on the forum was the most important way of providing the caregivers with knowledge of their situations, the illnesses of their spouses, research about healthcare, practical information about healthcare services or social security benefits. A caregiver could ask for information, such as “Do we have to renew a companion certificate?” A call centre nurse could respond, “I have heard that somebody above 70 years old has no companion certificate. Do some of you have the same experience? However, I will get in contact with the Social Welfare Office next week to explore if it’s age limited” (Call centre nurse).

In the second interview, the call centre nurse noted that she had improved her method of using the forum. “The content of the information is better now, and I have challenged caregivers to read and to be active in debates. I know they like it because they have told me” (Call centre nurse).

However, the nurses stated that building a relationship and a sustainable network would take time. Many caregivers had demanding days with their sick spouses and could not take any more time at the present. The nurses understood and respected that some caregivers did not want to use all parts of the service package or that they would take one thing at a time. On the one hand, the nurses wanted the caregivers to use the web camera as soon as possible. On the other hand, they did not want to force someone to use the camera. However, they tried to urge the person carefully and respectfully stimulate him or her to communicate via the web camera. To accelerate the caregivers’ adaptation to using all parts of the support service, the nurses organised a peer support system to follow-up with the new caregivers. The most important aspect was for the caregivers to become familiar with whatever part of the support system they appreciated the most. The nurses also argued that it was essential for them to know the caregivers’ situations well, so they could provide the necessary information and support and the opportunity for the caregivers to try something new.

Another demanding process for the nurses involved the caregivers’ variable activities in using the written dialogue on the forum. Some of the caregivers were posting messages just after becoming participants in the network, while others were just reading and remained observers. Two of the nurses reflected on the phenomenon:

Of course, we do want activity on the forum from each caregiver though it is a right for everyone to develop, as he wants for his own sake and situation. Someone appreciates social meetings and not computer communication, while others who have little opportunity to leave their homes prefer to use both the camera and the forum (Municipality nurse).

In my group, more of them are complaining about the content of the forum, but they do not do anything about it. Then, I tell them to be active contributors themselves, to make the agenda and ask for the others’ points of view. However, they are holding back. I can understand slowness, but I encourage activity (Municipality nurse).

The nurses accepted that caregivers might be worn out but tried to invite the observers to be active participants. In this way, they balanced the caregivers’ personal needs and respectfully tried to strengthen their competence in caring for themselves by using the resources in the network, such as support from the nurses, the experiences of the other caregivers and factual information.

### Supervision

Supervision seemed to be an important way of enhancing the caregivers’ ability to be aware of their own needs. Primarily, the call centre nurse did not provide medical supervision but advised the caregivers to visit a doctor if she observed something unusual during the web-camera dialogue. The supervision mostly concerned healthcare-related questions and how to obtain social services to ease the caregivers’ everyday life.

Challenges experienced by the caregiver in the relation to the patient were often issues in camera dialogues. The outcome of such dialogues varied. Some found it hard to follow the advices and guidance they received, and could react with despair as well as anger. It made the nurses at the call centre sad, to watch caregivers’ suffering while they would not apply for a respite stay for their spouses and some days off for themselves. One of the call centre nurses commented that she had challenged a caregiver several times to apply for a respite stay. This person reacted with anger, but he still complained about his situation in their web-camera dialogue.

Many of the caregivers were not used to applying for healthcare services, social security and welfare or filling out application papers. A call centre nurse knew the healthcare system very well and possessed the competence to provide practical help as well as to supervise caregivers in handling problematic situations. Caregivers often used the forum to ask questions related to problematic situations and to receive guidance in the healthcare system. In such cases, the call centre nurse described how she further tried to support them; for example, she said, “I will explore this for you on Thursday and write you an answer as quickly as possible. The taxi service should be a predictable system for you, so I will find out about their practice” (Call centre nurse).

## Discussion

A classic asymmetric patient–health personnel relationship is well known from nursing theory and can be exemplified by the supportive-educative nursing system described by Orem et al. [16]. Easier accessibility to ICT-based information has resulted in more people wanting to be more involved and collaborate in their own healthcare, and they are no longer willing to accept the classic, asymmetric relationship between patients and health care personnel [40]. The nurse–caregiver relationship in the CMC network described in this study indicates a possible change in the roles of nurses and caregivers from a classic asymmetric patient (caregiver)–health personnel relationship to a more symmetric relationship. The results suggest that the nurse–caregiver relationship may have altered, more or less in the same way as the classic asymmetric patient–health care personnel relationship has been modernised [41]. However, we have not found comparable studies that support our assumption regarding the nurse–caregiver relationship, which may indicate that this assumption might be relatively new knowledge.

Travelbee [17] points out the importance of the therapeutic use of the self with a combination of an educated mind and heart to attain the best outcome for the other. Such actions indicate a keen interest in humans and are fundamental elements of relational competence in developing a profound relationship [42]. Relational competence is an important quality to possess or acquire when building a nurse–caregiver relationship [16, 17]. This study showed nurses with relational competence who wanted to use their nursing agency for the maximum benefit of caregivers. This was demonstrated in situations where personal characteristics appeared; for example when they shared a beautiful view with the caregiver via web-camera communication and told each other stories from their daily lives or how they shared a sense of humour. Their professional qualities appeared in the way they cared for each individual, such as their awareness of the importance of listening to a person and the ways in which they organised the telecare service.

The nurses found that the use of the web camera furthered their ability to engage in face-to-face communication with the feeling of visiting a person in his or her private home. The appearance and the sounds of the caregiver offered the nurses a unique possibility to observe and feel the caregiver’s situation in their hearts. This allowed an atmosphere of confidential and honest communication. Such findings are supported by other studies [43–45]. However, Pols calls attention to the fact that the nurse usually is in an office, and the other person is at home. This may give some people the feeling of having their privacy invaded if they lack confidence in the nurse [46]. A required attitude, as suggested in nursing research, is therefore to arrange for privacy and confidentiality in web-camera communication when nurses provide care for individuals [26].

This study demonstrates that empathy emerged through the way nurses listened and tried to understand the caregivers’ situations. Spurkeland [42] states that an important ingredient in relational competence is emotional maturity, which addresses empathic and emotive intelligence. Delivering telecare entails a relational contact between the recipient and the moderator of the services to enable the health care personnel to carry out and convey such services. This interaction is a dynamic process wherein health care personnel have a particular responsibility to make themselves deserving of the recipients’ trust [19]. In our study, the nurses were aware of the possibilities and the limitations of this type of communication and stated that ethical thinking about the best interaction with caregivers should underpin how they used it. According to recent research [26], nurses need competence in communicating empathy, support and encouragement, both verbally and non-verbally, when using CMC. Nurses have the opportunity to use themselves therapeutically in a telecare setting so that their knowledge and attitudes can be transferred via a virtual setting [47]. One study [26] describes that a nurse’s attitude during a video conference ought to be characterised by ethical correctness, such as honesty, confidentiality and personal and professional integrity. Oliver and Dimiris [48] identified that caregivers recognised empathy in the eyes of a nurse. This may be associated with Travelbee’s theory [17] about using one’s self therapeutically, although she was not thinking of this attitude in a virtual context. This knowledge is crucial and informs healthcare practitioners that it is possible to transmit empathy from one person to another with the use of a web camera.

The use of written messages in web forums has limitations, depending on how nurses can obtain information about challenges and health problems and how they can pass on care to persons. There is also a risk that messages can be taken out of context and misunderstood [47]. This reduced possibility of obtaining information gives the nurse a different starting point for data collection, data assessment and communication of care and support [44]. However, Sullivan [49] claims that nurses have a unique opportunity to gain real insights into and understanding of caregivers’ perspectives and concerns by observing their dialogue in an online support group. Several studies describe how the flexibility of the network provides moderators with insights when the caregivers lack adequate and exhaustive knowledge to fulfil their need for obtaining information and giving advice to each other [24, 50, 51]. Studies indicate that information and education have been insufficient for individuals who have extreme challenges in their lives [50, 52]. In demanding situations, people need to develop their self-care agency because of their limitations with regard to knowing, judging and decision making [16]. In such situations, the moderator of a telecare service group has the possibility to assess in what way the supportive-educative method should be used for individual or group intervention [53]. Consequently, the telecare service offers nurses the possibility to interpret messages and to perceive whether individuals are in specific need of supervision.

In our study, some participants had an ambivalent attitude to the use of technology. Care recipients have different needs and capacities. According to both Orem et al. [16] and Travelbee [17], nursing prudence is an important quality that enables nurses to consider how care might best be provided to an individual. Reservations regarding the use of technology in connection with health care services have been reported in a number of studies until recently [54–56]. Virtual online consultation is still sparsely used even though its application is increasing in cases of patients with chronic illnesses [57, 58]. Telecare has often been accepted in combination with face-to-face health care services [59–63]. In this respect, the moderator’s role seems to be extremely important in terms of assessing whether face-to-face healthcare service, telecare or a combined service is suitable for the person in need of care [53, 64]. Related to our study, the telecare service comprised both virtual communication and information and face-to-face meetings with peers of caregivers and the nurses. This type of flexible telecare service might be important in cases in which people do not want to use the technology but are in need of supervision.

Orem et al. [16] claim that the purpose of nursing is to support people in the prevention of poor health and to care for those who are incapable of maintaining self-care, such as caregivers. Strengthening the caregivers’ ability to master their situation was an important part of the supervision. By using the supportive-educative nursing system and by virtue of their professional knowledge, nurses have the ability to choose an appropriate method to assist individuals in need of care. Orem, Taylor, and Renpenning [16] also claim that technology could bond people together in therapeutic relationships, which can help maintain individual integrity and lead to personal development despite illness and frailty. Lundberg’s study [21] underscores that older caregivers of persons with dementia experience close personal relationships with the staff at the call centre when a web camera is used. With the same staff connected to the patient in the long term, a trusting relationship based on intimate feelings is also fostered in patient–personnel relationships in palliative care [65]. To get more knowledge of how to establish positive virtual relationships, more studies about long-term relationships between users and health care personnel are recommended [44, 46, 47]. This study, based on long-term telecare service, is in line with this recommendation. The results of the study indicate that it is possible to establish a positive relationship in CMC for long-term services. In addition, the study describes features of this type of virtual relationship. Recent research states that studies focusing on this topic are still sparse, although the number is increasing [20, 57, 58].

### Limitations

The study has a small sample size. Two of the experienced nurses at the call centre finished their jobs soon after their first interview. The lack of their second interview could have influenced the analysis and the results. Neither the transcripts of the interviews nor the description of the findings were returned to the participants for comments, which might have influenced the way the results were described.

Because the researcher lacked access to the web forum to extract excerpts from it, the nurse in charge of the service did so. This might have resulted in certain discussions being overlooked. However, the data were collected over a two-year period in an attempt to capture continuing discussions. By doing so, we could create a broad picture of the types of nurse–caregiver dialogues.

The purpose of this study was to capture nurses’ perceptions of how they provide care and support via a web-based health care service. However, we realize that if we had included the caregivers’ experiences too, a more complementary picture would have emerged. This might have provided insight into what impact this form of communication/technology had on nurses’ and caregivers’ roles and levels of commitment.

## Conclusion

The nurse–caregiver relationship described by the nurses indicated a possible change from a classic asymmetric relationship to a more symmetric type. The nurses were striving to provide excellent support and care at a distance through CMC. The dialogues with the caregivers were characterised by closeness as well as empathy. Knowing that the caregivers were in stressful situations, the nurses endeavoured to enhance the caregivers’ knowledge, skills and competence. To strengthen the caregivers the nurses provided accessibility and supervision.

From the perspective of nurses, this study reveals more knowledge of long-distance dialogues and interactions between nurses and caregivers. It also contributes to bridging the gap between technologies and nursing care as potential conflicting dimensions.

The study indicates that also the social dimension of caring might be met when using this kind of technology in nursing. This knowledge gives rise to questions such as how essential a physical presence might be in different care situations. Web-camera communication appears to be a kind of interaction that makes it possible for nurses to provide a sense of closeness and express empathy, respect and care; values that are traditionally associated with nursing.

Findings in this study increase insight into how technology may change nursing and, accordingly, health care services. Such insight is essential for politicians, leaders in the health care service, and those who practice and teach health care. This is crucial to ensure that the technology is utilized for the benefit of the patient and society.

However, the study also indicates that the use of technology in several ways challenge the relationship between the caregivers and the nurses. Thus, more knowledge is required of the safety and maintenance of ethical principles regarding the use of this technology. Further research is also needed regarding caregivers’ experiences of long-distance dialogues with nurses.

## Data Availability

The datasets generated and/or analyzed during the current study are not publicly available due to the limited number of participants. The Regional Committee for Research Ethics in Norway required that the interviews and the excerpts from the web forum to be kept in locked files that were only accessible by the authors.

## Abbreviations

CMC: Computer-mediated communication
ICT: Information and communication technology
